# Pseudogenes and Their Genome-Wide Prediction in Plants

**DOI:** 10.3390/ijms17121991

**Published:** 2016-11-28

**Authors:** Jin Xiao, Manoj Kumar Sekhwal, Pingchuan Li, Raja Ragupathy, Sylvie Cloutier, Xiue Wang, Frank M. You

**Affiliations:** 1 Morden Research and Development Centre, Agriculture and Agri-Food Canada, Morden, MB R6M 1Y5, Canada; xiaojin@njau.edu.cn (J.X.); sekhwal@gmail.com (M.K.S.); pingchuan.li@agr.gc.ca (P.L.); 2 Department of Agronomy, Nanjing Agricultural University, Nanjing 210095, China; xiuew@njau.edu.cn; 3 Department of Soil Science, University of Saskatchewan, Saskatoon, SK S7N 5A8, Canada; 4 Department of Plant Science, University of Saskatchewan, Saskatoon, SK S7N 5A2, Canada; rajaragupathy@gmail.com; 5 Ottawa Research and Development Centre, Agriculture and Agri-Food Canada, Ottawa, ON K1A 0C6, Canada; sylvie.j.cloutier@agr.gc.ca

**Keywords:** pseudogenes, processed, duplicated, bioinformatics tools, plants, genome-wide

## Abstract

Pseudogenes are paralogs generated from ancestral functional genes (parents) during genome evolution, which contain critical defects in their sequences, such as lacking a promoter, having a premature stop codon or frameshift mutations. Generally, pseudogenes are functionless, but recent evidence demonstrates that some of them have potential roles in regulation. The majority of pseudogenes are generated from functional progenitor genes either by gene duplication (duplicated pseudogenes) or retro-transposition (processed pseudogenes). Pseudogenes are primarily identified by comparison to their parent genes. Bioinformatics tools for pseudogene prediction have been developed, among which PseudoPipe, PSF and Shiu’s pipeline are publicly available. We compared these three tools using the well-annotated *Arabidopsis thaliana* genome and its known 924 pseudogenes as a test data set. PseudoPipe and Shiu’s pipeline identified ~80% of *A. thaliana* pseudogenes, of which 94% were shared, while PSF failed to generate adequate results. A need for improvement of the bioinformatics tools for pseudogene prediction accuracy in plant genomes was thus identified, with the ultimate goal of improving the quality of genome annotation in plants.

## 1. Introduction

In 1977, Jacq et al. discovered in *Xenopus laevis* a truncated copy of a 5S rRNA gene with a compromised function, which they termed a “pseudogene” [1]. Pseudogenes have since been found to be ubiquitous in genomes [2]. A pseudogene is generally defined as a defective paralogous copy of a functional gene (“parent gene” or “cognate gene”) that has lost its capacity to produce a functional RNA or protein. Pseudogenes resemble their parent genes in DNA sequences but display disabling features such as absence of a promoter or deleterious mutations, resulting in internal premature stop codons or frameshifts that impair their transcription or translation [3]. Two major types of pseudogenes are categorized based on the mechanism of origin: processed pseudogenes, also termed retro-pseudogenes and duplicated pseudogene or unprocessed pseudogenes [4]. In the past, pseudogenes were generally considered as evolutionary dead-ends [5]. However, evidence shows that some pseudogenes possess functions in gene regulation [6]. Identification of pseudogenes may improve genome annotation and our understanding of genomes’ evolutionary history [7]. Most pseudogenes can be recognized through bioinformatics tools because they retain high similarity to their parent genes but possess identifiable disabling gene features. Pipelines for pseudogene discovery, such as PseudoPipe [8], Shiu’s pipeline [7], PSF (Pseudogene Finder) [9] and PPFinder (Processed Pseudogene Finder) [10], are publically available. To date, most researches on pseudogenes focus on mammals such as human and mouse. Herein, we summarize historical and recent progresses made in pseudogene studies, evaluate major pseudogene identification pipelines and assess their potential applications in plant genomes.

## 2. Origin and Formation of Pseudogenes

**Figure 1:**
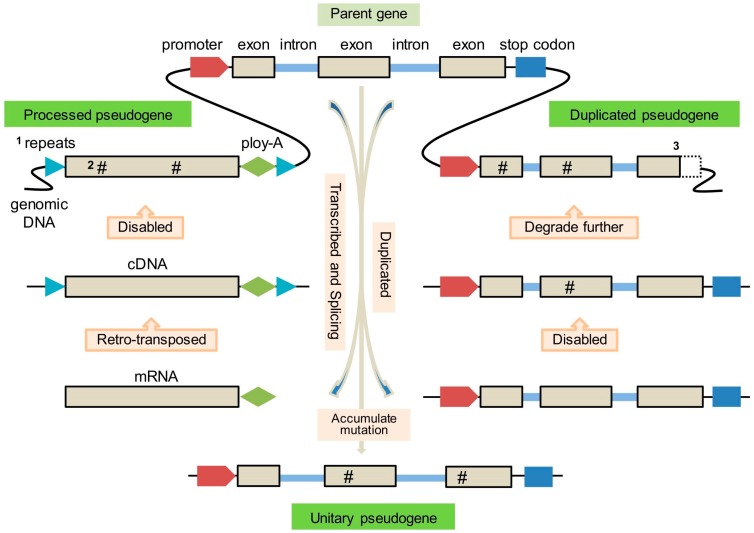
Illustration of pseudogenes formation. Note: **1**: Repeats associated with retro-transposition; **2**: The hashtag symbol (#) indicates deleterious mutations; **3**: The dashed box indicates truncation.

The formation of three types of pseudogenes is depicted in Figure 1. The pseudogene derived from a retro-transposition event, whereby the gene is transcribed into mature messenger RNA (mRNA), which is then reverse-transcribed into DNA and inserted elsewhere in the genome, is called a processed pseudogene (PPG) or retro-pseudogene [11]. The pseudogene arising by a duplication event, whereby the duplicated copy of a functional/parent gene acquires deleterious mutation and ultimately loses original protein-coding capacity, is known as a duplicated pseudogene (DPG) or unprocessed pseudogene [12]. An additional type, termed unitary pseudogene (UPG), has also been proposed and resembles a loss-of-function gene that may not have been duplicated before becoming disabled [13]. This type of pseudogene is similar to an unprocessed pseudogene but its paralogous counterpart is not found.

### 2.1. Processed Pseudogenes

This type of pseudogene is defined as “processed” due to its apparently altered features when compared with the parent gene. As it was copied from a mature mRNA, a PPG lacks a 5′ promoter sequence and intron(s) but exhibits a 3′ poly-A tail. The insertion site is flanked by direct repeats, a characteristic feature of transposable element (TE) insertions (Figure 1) [14]. Some PPGs show truncation at the 5′ end relative to the parent sequence that may be due to the low processivity of reverse transcriptase (RT), activity of RNAse H or defects of retro-transposition [15].

The retro-transposition responsible for the generation of PPGs is a fairly common event, and, as such, PPGs are abundant in mammalian genomes. The process is mediated by autonomous and non-autonomous retrotransposons, which are rich in current genomes and often represent the main component of nuclear DNA. For example, in maize they account for 49%–78% [16], in wheat 68% [17] and in human 42% of the genome [18]. PPGs resemble “non-autonomous retrotransposons” that were derived from gene retro-transposition events through hijacking of the retro-transposition machinery by host mRNA transcripts, mediated by, for example, long interspersed nuclear elements (LINEs or L1s) in human [19], and *Ty1* elements in *Saccharomyces cerevisiae* [20]. The abundance of PPGs may be correlated with retro-transposition activities in genome evolutionary history. Therefore, the number of PPGs varies across organisms. In the human genome chromosome 22, PPGs represent ~82% (110 out of 134) of the pseudogenes; this abundance may be due to a burst of retro-transposition activity occurring in the primate lineage approximately 40 million years ago (MYA) [21]. In plants, however, PPGs represent a much smaller percentage as illustrated by rice, in which approximately 23% (189 out of 816) of pseudogenes are PPGs [22].

The retro-transposed DNAs derived from mature mRNA usually lack the upstream promoters and are “pseudo” from the onset, i.e., upon insertion. In rare cases, these PPGs hitchhike existing genes [23], contributing additional exons.

### 2.2. Duplicated Pseudogenes

**Figure 2:**
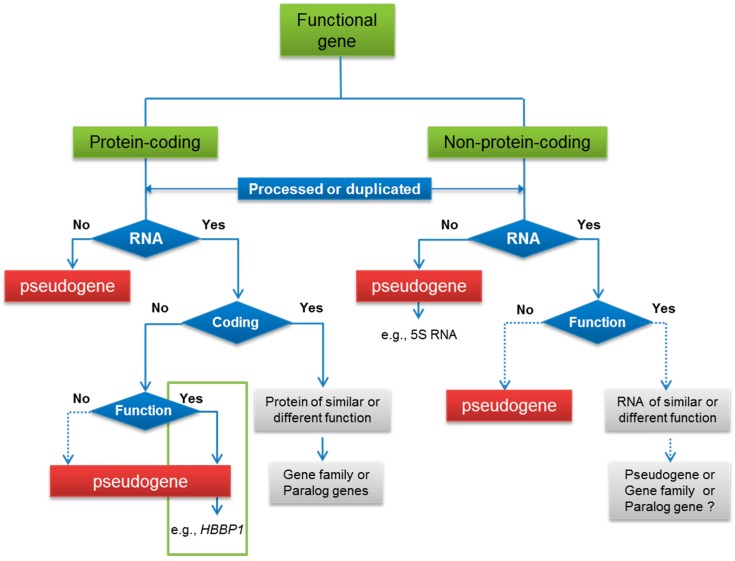
The emergence of processed or duplicated pseudogenes. Functional pseudogenes are indicated in the green framework. Solid arrows represent reference supports for the paths while dotted arrows show predicted paths. *HBBP1*: *Hemoglobin Subunit Beta Pseudogene 1*.

Compared with PPGs, DPGs preserve gene-like features such as promoters, original exon-intron structure and C-phosphate-G (CpG) islands [24], but have lost the ability to transcribe or encode RNA (e.g., ribosomal RNA pseudogene), or to code for proteins as a consequence of disabling mutations resulting in premature stop codons or frameshifts (Figure 2). Compared to PPGs, DPGs are therefore “unprocessed” (Table 1). However, the term “unprocessed” is not always accurate because some DPGs are truncated at the 5′ or 3′ ends [25].

As an evolutionary force for the generation of pseudogenes, gene duplication may result from the processes including (1) ectopic homologous recombination leading to an unequal crossing-over between misaligned homologous chromosomes sharing repetitive elements that may generate duplication-deletion products; (2) replication slippage—an error in DNA replication that can also produce duplications-deletions; and (3) whole genome duplication (WGD)—polyploidy, a phenomenon prevalent among plants where the entire gene content of a genome is duplicated through a single event [26]. Immediately after gene duplication, it creates genetic redundancy from the identical two copies. At this stage, no selective pressure should be against any loss-of-function mutation affecting either copy. This relaxation of purifying selection results in most instances the pseudogenization of one copy, as well as some amount of divergence, such as neo-functionalization and sub-functionalization for generating new functional paralog genes, which are subsequently maintained by purifying selection [27]. Overall, the tendency to become inactive pseudogenes is a general fate of duplicated genes [22].

Duplicated genes resulting from the above-described first two processes can result in a duplicated gene cluster where one or multiple genes will become disabled, thus creating pseudogenes. For instance, the functional β-globin and α-globin are the two protein chains that make up hemoglobin. β-globin is encoded by a cluster of genes on chromosome 11 derived from an unequal exchange. In the cluster of six *β-globin* genes, *Hemoglobin Subunit Beta Pseudogene 1* (*HBBP1*) is a pseudogene because it does not produce a functional or complete protein product due to several premature stop codons [28]. Disease resistance genes (*R*-genes) in plants are often clustered in genomes due to frequent unequal crossing-overs, a structural organization that enables neo-functionalization and rapid parallel adaptation to mutations in their pathogen counterparts. Aside from new functional genes, pseudogenes are also generated as a consequence of tandem duplications [29]. In potato, pseudogenes account for 41.6% of the total *R*-genes [30] and, in rice, the percentage reaches up to 55% [31].

WGD is usually followed by genome fractionation [32] leading to partial diploidization that reduces gene redundancy. The process results in (1) structural rearrangements [33]; (2) gene loss; (3) sub-functionalization leading to evolution of a novel gene functions [34]; and (4) functionless pseudogenes [34,35,36]. WGDs prevail in plants [37] and recent studies revealed that all angiosperms may have undergone two rounds of ancient WGD [38,39]. WGD-derived pseudogenes are not clustered with their parent genes as evidenced in rice and *Arabidopsis* [7,22].

### 2.3. Unitary Pseudogenes

An UPG is defined as an “unprocessed” pseudogene with no functioning counterpart (Figure 1), i.e., it is a single copy gene that is functionless [13], e.g., human and mouse *pbcas4* gene [40]. UPGs can also be categorized as a unique sub-family of DPGs [4] because failure to detect a parent gene does necessarily mean absence of a parent gene. An UPG could be an ancient DPG that has sufficiently diverged from its functional paralog such that its homology can no longer be detected [41]. However, most pseudogenes would likely be deleted [42] before they diverge to such an extent. As a result, UPGs account for only a small fraction of annotated pseudogenes, e.g., in the human genome only 76 UPGs were identified [13].

## 3. Functional Pseudogenes

Traditionally, pseudogenes have been considered “junk” DNA, in the sense that they were deemed evolutionary “dead-ends” or relics [43]. Pseudogenes may be the record of ancient genes and proteins. As such, the “pseudo” means functionless, either at an RNA [1] or protein level [28] (Figure 2). The discovery of “functional” pseudogenes [44] is changing this view. A typical example is the human β-globin pseudogene, which, while not coding for a protein, produces a variety of regulatory RNAs. Mutations in this pseudogene have been associated with blood diseases [45]. Another line of evidence for the functionality of some pseudogenes is that many are transcribed. In the human genome, 8.3% (876 out of 10,523) pseudogenes are transcribed, and, in Arabidopsis, approximately 20% (250 out of 1332) of all pseudogenes are transcribed [46,47]. Some pseudogenes are under purifying selection, which is another indicator for their potential function roles [48]. These observations are clues that some pseudogenes are not dead relics but may have novel functions important for adaptation and survival.

Most functional pseudogenes live on as RNA (Figure 2) and serve as sense or antisense regulatory transcripts to compete with transcription or interfere with translation of their parent genes, or produce small RNAs, including small interfering RNAs (siRNAs), micro RNAs (miRNAs) and Piwi-interacting RNAs (piRNAs), to silence cognate genes [6]. Some truncated pseudogenes, which possess essential upstream regulatory elements, could code for truncated peptides that function. Here are a few examples. Transcripts of pseudogene *PTENP1* (PPG group) compete with those of its cognate *PTEN* as target of miRNAs for degradation, functioning as a decoy competing for the binding between miRNAs and *PTEN* mRNAs, hence stabilizing *PTEN* mRNAs [49]. The nitric oxide synthase (NOS) pseudogene (DPG group) of snail *Lymnaea stagnalis* typically transcribes an antisense non-coding RNA, forming a stable RNA-RNA duplex with the functional NOS mRNA to reduce NOS protein production [50]. Transcripts of the ABC transporter gene *ABCC6* (PPG group) and its corresponding pseudogene *ABC6P1* could bind to produce siRNAs that, in turn, regulate *ABCC6* mRNA [51]. Pseudogene *connexin43* (ψ*Cx43*, PPG group) encodes a truncated 43 kDa protein functioning as part of the gap junction channels with the ability to inhibit cell growth [52].

Some pseudogenes, although appearing functionless, are biologically significant. In the *R*-gene family, pseudogenes may prime intragenic recombinations and gene conversion between alleles or paralogs, thus creating a mechanism for rapid formation of new *R*-genes [29]. Pseudogenes might represent DNA reservoirs that could be re-utilized to produce new genes.

The findings of functional pseudogenes build an ambiguous boundary between genes and pseudogenes. Thus, pseudogenes are suggested be redefined to resolve the intrinsic irony of functional pseudogenes [53]. “Exapted” pseudogenes or “ghost” pseudogenes were also proposed for functional pseudogenes, making them distinguished from putatively functionless pseudogenes [4,53]. Until now, only a few pseudogenes were proven to be functional. Insufficient attention has been devoted to this subject, and it is anticipated that the discovery of functional pseudogenes will accelerate with enhanced scrutiny.

## 4. Pseudogenes for Evolutionary Study

Pseudogenes are a window into the evolutionary past of genomes. In mammals, PPGs represent the dominant form of pseudogenes and are approximately three to four times more frequent than DPGs, implying that retro-transposition was important during the evolution of mammalian genomes [2,48]. In plants, however, the relative weight is reversed. In rice, for example, DPGs represent the majority of pseudogenes (75%), hinting at gene duplication being more important than retro-transposition during its evolution [22]. Using estimates of neutral nucleotide substitution rates, pseudogenes can be used to time evolutionary events [54]. For example, the age distribution of ribosomal protein (RP) pseudogenes is consistent with a decline in retro-transposition activity in the hominid lineage during the last 40 M years [55].

Pseudogenes help molecular biologists to uncover instances of gene births and deaths, just as the study of fossils tells paleontologists about the emergence and extinction of species [56]. For example, the finding of 47 human *cytochrome c* (*cyc*) pseudogenes presented an evolutionary record of the human *cyc* gene that showed an accelerated evolution in the primate lineage leading to the human [57].

Pseudogenes provide a powerful tool for phylogenetic studies to investigate genome evolution of closely related species. Conservation of pseudogenes was explored in human, chimpanzee, mouse, rat, dog and cow to study their descent from a common ancestor [4]. Because the age of conserved pseudogenes could be calculated, species differentiation time could be predicted.

PPGs are a reflection of the past expression of parent genes [58]. The functional genes that generated PPGs are predominantly highly expressed housekeeping genes or shorter RNAs such as genes encoding RPs [55].

## 5. Pseudogene Prediction

Pseudogene prediction is necessary in gene annotation. However, to identify a pseudogene by its non-functionality is difficult when it relies solely on laborious experimental evidence. As discussed above, the “no functional product” rule does not hold for all pseudogenes [53]. Therefore, bioinformatics approaches have been adopted for pseudogene prediction. Owing to the high similarity of sequences between “real” genes and pseudogenes, the homology-based approach has been predominantly used to search for pseudogenes [41,59].

Pseudogene detection relies on the alignment of parent genes to genetic regions beyond parent genes (or intergenic regions) to identify a parent-pseudogene homologous pair, followed by the detection of pseudogene-like features [2]. All pseudogenes are identified based on the three basic criteria: (1) similarity to their parent/functional genes (for both DPGs and PPGs); (2) disablements, such as deleterious mutations (mostly for DPGs and UPGs); and (3) defects in introns (specific for PPGs). Several algorithms and computer pipelines designed and implemented to predict pseudogenes focus mainly on the identification of DPGs and PPGs because they represent the majority of pseudogenes and can be predicted through such a bioinformatics approach (Table 2).

### 5.1. Establishment of a Set of Parent Genes

**Figure 3:**
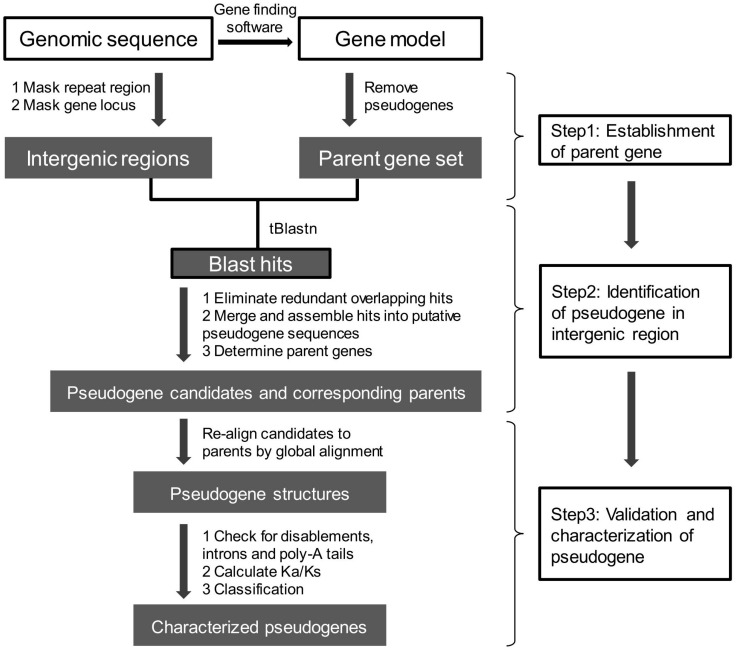
A flow chart of genome-wide pseudogene prediction methods.

The establishment of a set of accurately annotated genes that can be used as parent genes for detecting either DPGs or PPGs is the foremost important step, and is an integral part of every pipeline or tool developed to date (Figure 3). Evidence for identification of the parent genes includes protein sequences and ab initio gene prediction using gene finding software, often followed by supporting transcriptome evidence.

Functional proteins can be used directly as proxies for parent genes in search for corresponding pseudogenes. Using the 79 mammal RPs as parent genes, over 2400 RP pseudogenes and their fragments were identified in the human genome based on sequence-homology searches [55]. In *R*-genes, the consensus CC-NBS-LRR (CNL) and TIR-NBS-LRR (TNL) domain sequences were used as parent genes in search for other *R*-like genes where pseudo *R*-genes were defined as the subgroup being disabled [30,60,61,62,63]. For genome-wide pseudogene prediction, a comprehensive non-redundant protein sequence dataset is needed. To obtain accurate predictions, the protein database must be as complete as possible. The well curated ENSEMBL database [64] is a good source of functional proteins [24].

Information about parent genes can also be obtained from high quality gene annotations. Ab initio gene prediction programs such as N-SCAN [65], TWINSCAN [66], FGENESH [67], GeneMark.hmm [68], GENSCAN [69], GlimmerR [70] and some popular and comprehensive pipelines such as MIPS [71] and TriAnnot (specifically designed for wheat [72]) can build gene-like structural models from genomic sequences. However, the models may include both “real” genes and pseudogenes [47]. The quality of the annotation resulting from these programs was evaluated in maize. FGENESH provided the most accurate annotation [67]. Hence, irrespective of software, pseudogenes need to be removed from gene prediction sets to improve annotation. The most frequently used criterion is transcript evidence because the vast majority of pseudogenes are not transcribed [73,74]. Such annotated gene sets of mRNA evidence were used as parents to search for pseudogenes in the majority of this type of work reported to date [48]. However, if “real” genes and pseudogenes share a high degree of similarity, difficulties associated with their distinction remain. The gene annotation of most recently published genome assemblies is based on gene predictions and mRNA evidence, resulting in a large number of low-confidence genes where some are likely pseudogenes [75]. However, in rice, an alternative method was used for pseudogene identification [22]. High-confidence, well-supported genes were differentiated from low-confidence functional genes. Only high-confidence genes were used as parent genes in the search for pseudogenes in the low-confidence gene set, a step that was followed by pseudogene validation. The third strategy uses software to directly identify pseudogenes from an annotated gene set. For example, PPFinder is developed to identify and remove PPGs from N-SCAN gene prediction results of mammalian genomes, which substantially improves gene annotation [10]. PPFinder was not designed to ab initio predict pseudogenes from intergenic regions of a given genomic sequence. Similar to the transcriptome evidence to validate gene prediction, it also attempts to integrate pseudogene removal with gene prediction.

### 5.2. Pseudogene Identification from Intergenic Regions

Once the parent gene set is established, two main steps are taken: (1) the search for pseudogene candidates in intergenic regions of a genome based on homology; and (2) the examination of candidates for pseudogene-like features, including the disablements that describe deleterious mutations, including premature stop codons, frameshifts, indels and a lack of introns, the characteristic feature of PPGs. The former is conducted by tBLASTn analysis, a local sequence alignment of amino sequences against repeat-masked genome sequences, to obtain pseudogene candidate sequences, and the latter is accomplished through FASTA [76], Prot_map [9] or GeneWise [77] algorithms to achieve a refined global alignment that shows positions of disablement (Figure 3). For this purpose, pipelines such as PseudoPipe [8], Shiu’s pipeline [7] and PSF [9] have been developed.

#### 5.2.1. Identification of Candidate Pseudogenes and Their Parent Genes

The first step includes the localization of pseudogene candidate target regions, and determination of their parents. The tBLASTn algorithm is used with parent gene protein sequences as queries to identify pseudogene candidate regions on the repeat-masked intergenic DNA sequences. When gene regions are not pre-masked (as in PseudoPipe), BLAST hits corresponding to the regions of parent genes will be removed and the remainder will be retained as pseudogene candidate regions; when gene regions are pre-masked (as in Shiu’s pipeline and PSF), the BLAST hits corresponding to intergenic regions are acknowledged as target regions. Here, the redundant and overlapping BLAST hits need to be eliminated because the same DNA segment may be hit by either the same or different query protein(s). The hits are treated as follows: significant matching hits are retained (e.g., E-value < 1 × 10^−5^); the hits matching distinct queries are separated; for each query the hits are partitioned into disjoint sets for removal of overlaps. The “disjoint hits” resemble “pseudogene exons”. If adjacent “disjoint hits” match to the same query proteins, they are merged as a single entity based on their distances. Then, pseudogene structures are reconstructed. Additionally, the same region in a genome may be hit by multiple parent genes, including paralogs. In this case, the top hit of parents would be chosen so that a pseudogene has a unique parent.

#### 5.2.2. Pseudogenes Validation and Classification

After pseudogene candidates and their parents are established, candidates are re-aligned to the query parent proteins using a global alignment tool, such as tfasty (used in PseudoPipe and Shiu’s pipeline) and Prot_map (in PSF). The global alignment "forces" proteins to span the entire length of pseudogene sequences in a global optimization. The alignment poses a structure for pseudogene with potential internal stop codons, frameshifts and presence or absence of introns [9]. Therefore, pseudogenes can be validated for these disablement features and classified. Some pipelines conduct further verification by detecting poly-A tail signals to differentiate PPGs, because some parent genes have no introns [55].

The non-synonymous/synonymous substitutions rate (Ka/Ks), calculated from a pseudogene candidate and its percentage, may provide an additional evaluation for all pseudogenes but not for individual pseudogenes [22]. Pseudogenes are commonly acknowledged not to be under selection constraint. Thus, pseudogenes are expected to have a Ka/Ks ratio close to 1, while functional genes should have a Ka/Ks ratio much lower than 1 since replacement mutations are subjected to purifying selection. In rice, the pseudogene’s Ka/Ks distribution was log-normal with a geometric mean of 0.32, which, although lower than the expected 1, is significantly larger than the 0.14 value obtained for functional genes [22].

### 5.3. Comparisons of Bioinformatics Tools for Pseudogene Prediction in Plants

Pseudogenes have been predicted manually, semi-automatically or automatically in different studies. Table 2 summarizes some approaches and pipelines for pseudogene prediction. They generally go through the same three main steps illustrated in Figure 3. All approaches or tools in Table 2 were primarily applied in mammalian genome studies. Only PseudoPipe, Shiu’s pipeline and PSF are publicly available automatic pipelines for pseudogene prediction in intergenic regions of genomes. To determine whether these three tools can be efficiently applied to plant genomes, we used the well-annotated *Arabidopsis thaliana* genome, which has 924 known pseudogenes in the latest annotation version (TAIR10).

All sequence and gene annotation data of the *Arabidopsis thaliana* genome (TAIR 10) was downloaded from the Ensemblplants genome database (release-32, http://plants.ensembl.org). A total of 27,206 genes with protein sequences were used as a common input for parents to test all three tools mentioned above. The genome sequences were repeat-masked and exon coordinate files of parent genes on separate chromosomes were prepared for PseudoPipe. The repeat-masked genome sequences were further masked by substituting the parent gene regions with Ns, a necessary step for Shiu’s pipeline and PSF. The resulting sequences represent intergenic regions of the genome for pseudogene detection. PseudoPipe, Shiu’s pipeline and PSF were downloaded from their corresponding web sites (Table 2).

**Figure 4:**
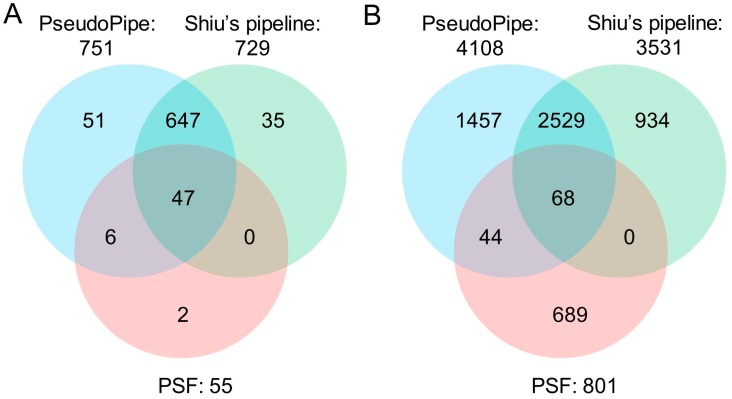
Comparisons of three bioinformatics tools for pseudogene prediction using the *A. thaliana* genome with its 924 known pseudogenes. (**A**) Identified known pseudogenes; (**B**) all identified pseudogenes.

A total of 4108, 3531 and 801 pseudogenes were predicted by PseudoPipe, Shiu’s pipeline and PSF, respectively, corresponding to 2550, 2317 and 604 parent genes (Table 3 and Table S1). To confirm whether a predicted pseudogene was the same as a known pseudogene, we calculated a percentage of overlap between predicted and known pseudogenes through their chromosome coordinates. When a predicted pseudogene overlapped uniquely more than 60% with a known pseudogene, we declared them to be the same. According to this criterion, 751 (81.3%), 729 (78.9%) and 55 (6.0%) out of the 924 known pseudogenes of *Arabidopsis* were identified by PseudoPipe, Shiu’s pipeline and PSF, respectively (Figure 4A, Table 3). The three tools combined identified a total of 794 (85.9%) known pseudogenes including 57, 35 and 2 known pseudogenes unique to a single of them, respectively. A total of 130 known pseudogenes could not be predicted with these tools. These pseudogenes were further investigated and it was found that 68 had no homologous genes (data not shown), implying that they may belong to UPGs, which are not predicted using these bioinformatics tools. After removal of UPGs from the known gene set, both PseudoPipe and Shiu’s pipeline identified a higher percentage of known pseudogenes, i.e., 87.7% and 85.2%, respectively. They predicted 694 common known pseudogenes (94% of similarity between them), demonstrating that two tools are similarly effective in finding “real” pseudogenes. PSF did not produce adequate results because it considerably under-predicted pseudogenes and corresponding known pseudogenes. Further comparisons among the three tools using all identified pseudogenes in *Arabidopsis* showed similar results as above with 2529 common pseudogenes and a similarity of 68.0% between PseudoPipe and Shiu’s pipeline (Figure 4B). In addition, of the 4108 pseudogenes identified by PseudoPipe, 629 (15.3%) were classified as processed, 1133 (27.6%) as duplicated or unprocessed while the remaining 2338 (58.2%) were deemed “fragments” due to their short size compared to their parent’s. PseudoPipe outperformed Shiu’s pipeline because the latter lacked classification information.

PseudoPipe and Shiu’s pipeline identified ~80% more pseudogenes than the known number of *Arabidopsis* pseudogenes. It is difficult to determine whether these pseudogenes are “false-positive” or candidates due to the limited number of studies to address the annotation of pseudogenes in *Arabidopsis* as well as other plant genomes.

## 6. Pseudogenes in Plants

Pseudogenes have been reported mostly in human, mouse and other mammalian genomes [81,82,83]. Limited efforts have been put into pseudogene studies in plants. In rice, Thiband-Nisseu et al. identified 1439 pseudogenes from a gene complement of 22,033 genes [22]. A total of 28,330 and 4771 pseudogenes in whole-genome intergenic regions of rice and *Arabidopsis*, respectively, were also reported [7]. In these studies, similar pseudogene prediction procedures to those described above were used but these were supplemented with manual annotations. Pseudogenes were also identified in *Triticum* species, which were defined as low confidence loci [84,85,86] based on conserved sequence comparison to wheat full length cDNAs and its reference plant genome. The method used was rather imprecise compared to those used in human and the model plants. Overall, automatic and accurate prediction pipelines for plants remain much needed to accelerate research in this area and provide reasonable prediction accuracies.

## 7. Conclusions

Pseudogenes are gene copies that are derived by duplication or retro-transposition from functional genes and therefore retain much of the original sequence and structure. They accumulate mutations in coding sequence such as frameshifts and premature stop codon that may impair their transcription or translation. However, we would like to mention that the definition of pseudogene is evolving over time based on the emergence of new evidence about their expression and potential role in regulation. Thus, pseudogenes can be basically defined as gene paralogs containing critical defects in their sequences, and can be predicted using a bioinformatics tool. Whether they have functions or not must be determined by laboratory work. Two similar computing pipelines PseudoPipe and Shiu’s pipeline can be borrowed for plant pseudogene prediction. Advanced bioinformatics tools remain needed to improve the accuracy of pseudogene prediction and genome annotation in plants.

## Figures and Tables

**Table 1:** Comparison between processed and duplicated pseudogenes.

	Processed Pseudogenes	Duplicated Pseudogenes
1	Arise from mRNA that was reverse-transcribed and re-integrated into the genome	Arise from gene duplication
2	Lack of non-coding intervening sequences: introns and promoters	Possess promoters, exon-intron structure and other upstream regulatory sequences
3	Possess a poly-A tail at 3′ end	No 3′ poly-A tail
4	Possess flanking direct repeats associated with TE insertion sites	No flanking direct repeats
5	Mostly present at different loci from its parent genes	Some are present as a cluster with their parent gene as a consequence of tandem segmental duplication
6	Have 3′ or 5′ truncations	Have 3′ truncations
7	Generally shorter	Comparatively longer

**Table 2:** Bioinformatics pipelines or approaches for predicting pseudogenes.

Pipeline/Method	Input Data	Brief Description	Availability	* Ref.
Harrison’s Approach	Protein and genome sequence, annotation information	Using protein sequences to find pseudogenes in intergenic regions by FASTA alignment; refinement of alignments for validation and classification	Method, not a pipeline tool	[78]
Sakai’s Approach	cDNA and genome sequence	Using cDNA to search and extract corresponding regions from genome sequence by BLASTn; realignment of cDNAs to extract sequence; using est2genome for classification	Method, not a pipeline tool	[79]
PPFINDER (Processed Pseudogene Finder)	Gene model and cDNA database	Using cDNA as evidence to determine parent genes in gene models; using parent genes to detect locus missing introns by BLASTN search; removing false candidates	http://www.mybiosoftware.com/	[10]
PseudoFinder	Functional genes and genome sequence	Finding homologues of functional genes in a genome; classification into either pseudo or functional categories using Support Vector Machines (SVMs) based on a combination of features by BLASTz analysis	Not available online	[48]
RetroFinder	GenBank mRNA and genome sequence	Alignment of mRNAs from GenBank to genome sequence by BLASTz; detection of biological features; heuristic weighting for known PPGs	Not available online	[48]
GIS-PET (Gene identification signature-paired end tag) method	mRNA and genome sequence	Using 5′ and 3′ paired-end-tag (PET) of mRNAs to select candidates based on homology; using the shortest candidate to search the genome by BLAT	Method, not a pipeline tool	[80]
PseudoPipe	Genome sequence (repeat marked), parent proteins and their exon coordinates	Using protein sequence to find pseudogenes in repeat-masked intergenic regions by tBLASTn; realignment of candidates to corresponding parent(s) by FASTA to validate and classify pseudogenes	http://www.pseudogene.org/pseudopipe/	[8]
Shiu’s pipeline	Parent proteins and genome sequence (repeat-masked and intergenic)	Using protein sequence to find pseudogenes in repeat-masked intergenic regions by tBLASTn; realignment of candidates to corresponding parent(s) by FASTA to validate pseudogenes. Similar to PseudoPipe	http://shiulab.plantbiology.msu.edu	[7]
PSF (Pseudogene Finder)	Same as Shiu’s pipeline	Using protein sequence to find pseudogenes in repeat-masked intergenic regions directly by Pro-map to detect disruption events and classify pseudogenes	http://molquest.com/	[9]

* Ref.: Reference.

**Table 3:** Comparison of thee bioinformatics tools employed for pseudogene prediction using the *Arabidopsis thaliana* genome sequence with its 924 known pseudogenes.

Tool	No. of Total Pseudogenes Identified	No. of Parents Associated	No. of Known Pseudogenes Identified	Known Pseudogenes Identified (%)
PseudoPipe	4108	2550	751	81.3
Shiu’s pipeline	3531	2317	729	78.9
PSF	801	604	55	6.0

## Supplementary Materials

Supplementary materials can be found at www.mdpi.com/1422-0067/17/12/1991/s1.

## Abbreviations

PPG	processed pseudogene
DPG	duplicated pseudogene
UPG	unitary pseudogene
WGD	whole genome duplication
TE	transposable element
ORF	open reading frame
RT	reverse transcriptase
mRNA	messenger RNA
miRNA	microRNA
siRNA	short interfering RNA
piRNA	piwi-interacting RNA
RP	ribosomal protein
MYA	million years ago
NOS	nitric oxide synthase
Ka/Ks	non-synonymous/synonymous substitutions rate
CpG	C-phosphate-G
